# Prevalence and Phenotypic and Genotypic Resistance Mechanisms of Multidrug-Resistant *Pseudomonas aeruginosa* Strains Isolated from Clinical, Environmental, and Poultry Litter Samples from the Ashanti Region of Ghana

**DOI:** 10.1155/2021/9976064

**Published:** 2021-06-15

**Authors:** Hayford Odoi, Vivian Etsiapa Boamah, Yaw Duah Boakye, Christian Agyare

**Affiliations:** ^1^Department of Pharmaceutical Microbiology, School of Pharmacy, University of Health and Allied Sciences, Ho, Volta Region, Ghana; ^2^Department of Pharmaceutics, Faculty of Pharmacy and Pharmaceutical Sciences, Kwame Nkrumah University of Science and Technology, Kumasi, Ghana

## Abstract

**Background:**

Antibiotic resistance in bacteria is a major global health challenge. Reports on the prevalence of multidrug-resistant *P. aeruginosa,* a common pathogenic bacterium implicated in nosocomial infections and poultry diseases, are limited in Ghana. This study therefore sought to determine the prevalence of *P. aeruginosa* from hospitals, poultry farms, and environmental samples from the Ashanti region of Ghana. *Methodology*. Stool, urine, and blood samples from 364 patients from two hospitals *in the Ashanti region of Ghana* were randomly sampled*. P. aeruginosa* was isolated and confirmed using routine selective media and PCR-based *oprL* gene amplification. The Kirby-Bauer disk diffusion method employing EUCAST breakpoint values was used to identify multidrug-resistant strains. The occurrence of common antibiotic inactivating enzymes and resistance encoding genes and the assessment of strain efflux capacity were investigated with double disc synergy test (DDST), imipenem-EDTA synergy test, phenylboronic acid test, D-test, routine PCR, and ethidium bromide agar-cartwheel method.

**Results:**

A total of 87 (9.7%, *n* = 87/900) *P. aeruginosa* isolates were confirmed from the samples. 75% (*n* = 65/87) were resistant to more than one group of antipseudomonal agents, while 43.6% (*n* = 38/87) were multidrug-resistant (MDR). High prevalence of extended spectrum *β*-lactamases (84.2%), metallo-*β*-lactamases (34.1%), and AmpC inducible cephalosporinases (50%) was observed in the MDR strains. About 57.8% of the MDR strains showed moderate to very high efflux capacity. Class 1 integrons were detected in 89.4% of the MDR isolates but *β*-lactamase encoding genes (*bla*_*SHV*_, *bla*_*TEM*_, *bla*_*CTX-M*_, *bla*_*VIM*_, and *bla*_*IMP*_) were not detected.

**Conclusion:**

Surveillance of antibiotic-resistant strains of bacteria should be routinely conducted in clinical and veterinary practice in Ghana to inform selection of antibiotics for therapeutic use.

## 1. Introduction

The emergence and spread of multiple drug-resistant pathogenic bacteria is a major global health concern [1]. The natural resistome of bacteria and human influences on antibiotic use are contributory factors in the evolution of resistant bacteria [2]. The increased use of antibiotics in clinical, environmental, and agricultural settings has become pivotal in the selection and spread of resistant bacteria [3]. The evolution of resistance in bacteria may be due to mutational events introduced during bacteria replication and vertical transmission of genetic variants through generations in a particular bacteria strain [4]. Accessory genetic elements carrying antibiotic resistance determinants (plasmids, integrons, and transposons) may also be disseminated horizontally in bacteria leading to wide spread of resistance [5]. In Ghana, antibiotics are easily accessible for prophylactic and metaphylactic purposes as well as for growth promotion in animal husbandry [6]. Prescribing of large doses of broad-spectrum antibiotics, nonadherence to prescribed doses, and long durations of antibiotic treatments in district and regional hospitals in the country have enhanced the evolution of drug-resistant strains in pathogenic bacteria [7]. According to a study by Newman et al. [8], *Pseudomonas* species were the second most prevalent (14.0%) bacteria isolated during a six-month nationwide clinical surveillance study in Ghana. *P. aeruginosa*, a ubiquitous pathogen, is implicated in some nosocomial infections in patients [9]. It also causes pseudomoniasis, an opportunistic infection in poultry birds like chickens, turkeys, ducks, geese, and ostriches, where infection in eggs kills embryos [10]. *P. aeruginosa* is intrinsically resistant to many antimicrobial agents presenting clinicians with a great challenge during therapy. Several multidrug-resistant strains have been identified in many environmental niches from several countries [11]. *β*-lactam antibiotics, carbapenems, aminoglycosides, and quinolones play vital roles in the treatment of *P. aeruginosa* infections. However, multiple resistance of *P. aeruginosa* to these classes of antibiotics is on the surge. Resistance against these groups of antibiotics is influenced by varied resistance mechanisms. Predominant of these is the hydrolytic and inactivating activity of antibiotic degrading enzymes such as *β*-lactamases and aminoglycoside modifying enzymes [11], coupled with efficient efflux porins and external biofilm matrix. Mutations in the quinolone resistance determining region (QRDR) of DNA gyrase and topoisomerase IV alter the structural binding site of quinolones and thus confer reduced susceptibility. Reports on the prevalence of multidrug *P. aeruginosa* isolates and antibiotic resistance determinants in resistance-selection prone environments such as hospitals, animal farms, and sewages in Ghana are limited. The study therefore sought to determine the occurrence of *P. aeruginosa* strains and the prevalence of common antibiotic inactivating enzymes, mobile genetic elements (integrons), and resistance encoding genes in *P. aeruginosa* from selected poultry farms, hospitals, and market environments in Kumasi, Ghana.

## 2. Methods

### 2.1. Ethical Clearance/Approval

Ethical clearance for the study was obtained from the Committee on Human Research Publications and Ethics (CHRPE), Kwame Nkrumah University of Science and Technology (KNUST), Kumasi, Ghana. In addition, written consents were obtained from patients, farm managers, and workers and all participants in the study.

### 2.2. Study Sites, Subjects, and Sampling

**Figure 1:**
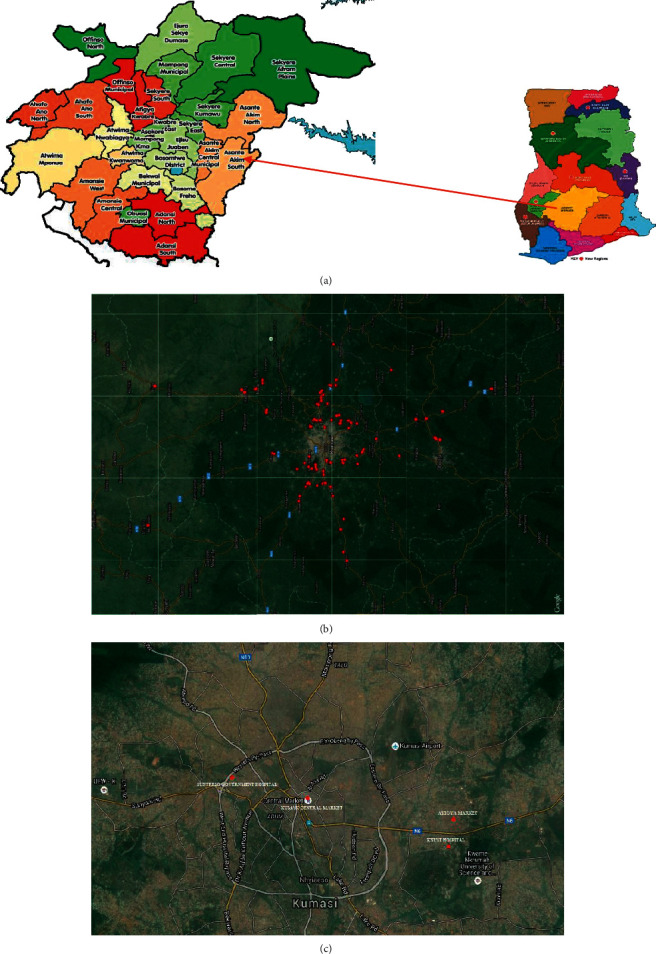
(a) Map of Ghana showing Ashanti region (study area) with detailed boundaries of all the districts. (b) Distribution of towns in the Ashanti region of Ghana where poultry farms were sampled. (c) Distribution of hospitals and markets in Kumasi where clinical and environmental samples were obtained.

The study was performed in Ashanti region in the central part of Ghana, located between 0.15–2.251 W and 5.50–7.46 N (Figure 1(a)). The region shares boundaries with five of the 16 political regions of Ghana. The region covers a total land area of 24,389 km^2^, representing 10.2% of the total land area of Ghana. 2 hospitals, namely, Suntreso Government Hospital and Kwame Nkrumah University of Science and Technology (KNUST) Hospital, 137 poultry farms (Figure 1(b)), 1 public market (Kumasi central market), and Ayigya town, all located in Kumasi in Ashanti region of Ghana (Figure 1(c)), were selected for the study. Kumasi central market was selected because the market has a high population of traders and is the point of sale for most agricultural products from most parts of the region. Stool, urine, and blood samples from 364 patients were randomly sampled from the two hospitals. A total of 276 poultry litter samples were collected from 137 poultry farms in the Ashanti region of Ghana. All the samples were collected between September 2015 and July 2016 as part of routine AMR surveillance [12]. Swabs of community-based latrines, market floors and tables, soil, and sewage were also carried out for *P. aeruginosa* isolation.

### 2.3. Isolation and Identification of *P. aeruginosa*

**Figure 2:**
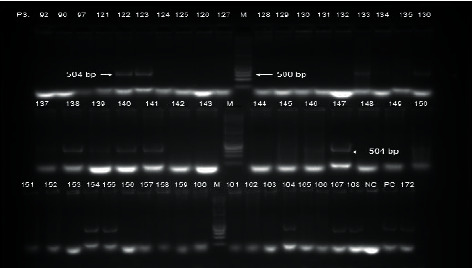
Gel electrophoretic image showing a 504 bp PCR amplicon of a peptidoglycan associated outer membrane lipoprotein gene (*oprL*) in *P. aeruginosa*. (M) DNA marker PC: positive control (*P. aeruginosa* ATCC 27853).

Bacteria in the various samples collected were revived in soybean-casein-digest broth (Thermo Fisher Scientific, Waltham, MA, USA) and isolated on cetrimide agar (Thermo Fisher Scientific, Waltham, MA, USA). Preliminary identification was then conducted through Gram staining test for catalase and oxidase activities, haemolysin production, and growth at 42°C. Production of pyocyanin, pyomelanin, pyorubin, and pyoverdine pigments was examined by culturing the isolates on *Pseudomonas* isolation agar (Alpha Biosciences, Baltimore, MD, USA). The isolates were confirmed by amplification of the species-specific outer membrane lipoprotein gene *oprL* (Figure 2) which provides confirmation for all phenotypes of this species [13]. With 0.6 *μ*L each of a 10 *μ*M forward primer [oprL-F (5′-ATG GAA ATG CTG AAA TTC GGC-3′)] and reverse primer [oprL-R (5′-CTT CTT CAG CTC GAC GCG ACG-3′)], polymerase chain reaction was carried out using a thermal cycler (GeneAmp, Thermo Fisher Scientific, Waltham, MA, USA) in a final volume of 25 *μ*L containing 2 *μ*L of DNA template, 12.5 *μ*L of GoTaq Master Mix (Promega, Madison, WI, USA), 0.75 *μ*L of a 0.5 mM magnesium chloride, and 8.55 *μ*L of nuclease-free water. The DNA template was initially denatured at 94°C for 5 min, followed by 35 cycles of denaturation at 94°C for 30 sec, annealing at 64°C for 30 sec, and extension at 72°C for 1 min. Finally, the products were extended at 72°C for 10 min. The PCR products were examined on a 2% w/v agarose gel at 60 V and visualized using a transilluminator (Fotodyne, Hartland, WI, USA).

### 2.4. Antibiotic Susceptibility Testing

Multidrug-resistant *P. aeruginosa* isolates were determined by determining the susceptibility of the *P. aeruginosa* isolates to selected antipseudomonal antibiotics using the Kirby-Bauer disk diffusion technique. The susceptibility tests were done in triplicate according to approved methods of the European Committee on Antimicrobial Susceptibility Testing [14]. To ensure a good representation of the isolates in the culture, about 5 to 7 well-separated colonies were picked and suspended in 5 mL sterile distilled water and vortexed at high speed until the suspension was uniform. The turbidity of the suspension of *P. aeruginosa* isolates was determined using a nephelometer already calibrated to 0.5 McFarland. The turbidity of the suspensions was then adjusted appropriately to 0.5 McFarland, either by the addition of more colonies or sterile distilled water. A sterile cotton swab was soaked in the inoculum and rotated twice against the inner side of the test tube to remove excess liquid. The swab was used to streak the entire surface of 20 mL Mueller-Hinton agar (Oxoid, London, UK) plate while rotating the plate at an angle of 60° with repeated streaking (three times in total). With the aid of a disk dispenser, eleven antibiotic disks from six different classes including piperacillin (PIP-100 *μ*g, Oxoid Ltd, Basingstoke, UK), ticarcillin (TIC-75 *μ*g, Oxoid Ltd, Basingstoke, UK), ceftazidime (CAZ-30 *μ*g, Oxoid Ltd, Basingstoke, UK), cefepime (FEP-30 *μ*g, Oxoid Ltd, Basingstoke, UK), aztreonam (ATM-30 *μ*g, Oxoid Ltd, Basingstoke, UK), imipenem (IPM-10 *μ*g, Oxoid Ltd, Basingstoke, UK), meropenem (MEM-10 *μ*g, Oxoid Ltd, Basingstoke, UK), ciprofloxacin (CIP-5 *μ*g, Oxoid Ltd, Basingstoke, UK), gentamycin (CN-10 *μ*g, Oxoid Ltd, Basingstoke, UK), levofloxacin (LEV-5 *μ*g, Oxoid Ltd, Basingstoke, UK), and ticarcillin/clavulanic acid (TIM-85 *μ*g, Oxoid Ltd, Basingstoke, UK) were used and incubated at 37°C for 24 h and the mean growth inhibitions and standard deviations calculated. Strains that were resistant to at least one agent from three or more antibiotic classes were identified as multidrug-resistant [15]. *P. aeruginosa* ATCC 27853 was used as a reference control strain.

### 2.5. Double Disc Synergy Test (DDST) for Extended Spectrum *β*-Lactamase (ESBL) Detection

The DDST is used for the detection of beta-lactamases that are inhibited by beta-lactamase inhibitors such as clavulanic acid [16]. 20 mL of Mueller-Hinton agar (Oxoid, London, UK) plates containing 200 *μ*g/mL of cloxacillin was inoculated by swabbing a 1.5 × 10^8^ cfu/ml standardized inoculum of *P. aeruginosa* on the surface of the agar using a sterile cotton swab. Amoxicillin-clavulanic acid (20/10) *μ*g disc was placed at the center of the inoculated media. Cefepime (30 *μ*g), ceftazidime (30 *μ*g), cefotaxime (30 *μ*g), ceftriaxone (30 *μ*g), imipenem (10 *μ*g), and aztreonam (30 *μ*g) discs were placed 20 mm from the central amoxicillin-clavulanate disc. The plates were then incubated at 37°C for 24 h. The presence of a “ghost inhibition zone” or a synergistic inhibition of any of the antibiotics towards the central antibiotic was recorded.

### 2.6. Imipenem-EDTA Synergy Test for Metallo-*β*-Lactamase (MBL) Detection

**Figure 3:**
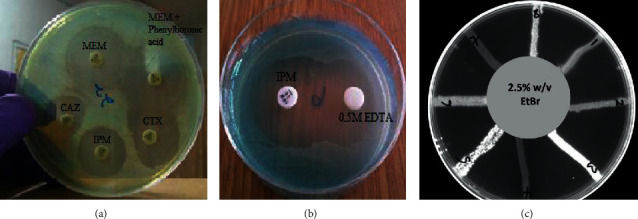
(a) Boronic acid test for detection of KPC-type carbapenemases (no synergy observed in the activity of meropenem in the presence of a KPC enzyme inhibitor-phenylboronic acid); D-test for detection of inducible AmpC *β-* lactamases (D-shaped inhibition zone of substrate antibiotic (ceftazidime and cefotaxime)). (b) Imipenem-EDTA synergy test for detection of metallo-*β*-lactamases production (enhancement of imipenem inhibition zone towards EDTA due to MBL inhibitory activity of EDTA). (c) Fluorescence of MDR *P. aeruginosa* on ethidium bromide incorporated Mueller-Hinton agar plates. IPM: imipenem; MEM: meropenem; CAZ: ceftazidime; CTX: cefotaxime; EDTA: ethylenediaminetetraacetic acid. EtBr: ethidium bromide.

EDTA, a polyaminocarboxylic acid, binds metal ions like zinc and inactivates metallo-*β*-lactamases that use zinc to break the amide bond in substrate antibiotics [17]. Imipenem-EDTA synergy test was performed according to the method described by Lee et al. [17]. Mueller-Hinton agar (20 mL) plates were inoculated with 5 × 10^8^ CFU/mL of the test organisms. An imipenem disc (10 *μ*g) was placed 20 mm from a blank disk containing 10 *μ*L of 0.5 M EDTA (Figure 3(b)). Enhancement of the zone of inhibition in the area between the imipenem and EDTA disks was considered as positive for metallo-beta-lactamase production.

### 2.7. Phenylboronic Acid Test for KPC Carbapenemase

Phenylboronic acid acts as an inhibitor of the hydrolytic activity of KPC carbapenemases and classes A and C *β*-lactamases [18]. *P. aeruginosa* suspension diluted to 0.5 MacFarland was swabbed on 20 mL Mueller-Hinton agar and two meropenem discs were placed 30 mm from each other. 20 mL of 20 g/L phenylboronic acid was added to the second meropenem disc and incubated at 37°C for 20 h (Figure 3(a)). *A* ≥ 5 mm increase in inhibition zone of the combined meropenem and phenylboronic acid disc compared to the meropenem disc alone indicated production of KPC carbapenemase enzyme by the *P. aeruginosa* strain [19].

### 2.8. D-Test for Detection of Inducible AmpC Beta-Lactamases

The D-test which incorporates an inducer of AmpC enzyme together with a substrate antibiotic as described by Dunne and Hardin [20] was used for the detection of AmpC *β*-lactamase production. An antibiotic disc inducing production of AmpC beta-lactamase enzyme (imipenem) was placed between two substrate antibiotic discs (ceftazidime and piperacillin-tazobactam) on an inoculated 20 mL Mueller-Hinton agar. The plate was incubated for 24 h at 37°C. The formation of a D-shaped inhibition zone around any of the substrate discs indicates the imipenem-mediated induction of the AmpC production and the subsequent inactivation of the substrate antibiotic by the *β*-lactamase.

### 2.9. Assessment of Efflux Pump Activity in *P. aeruginosa*

Ethidium bromide- (EtBr-) agar cartwheel method as described by Martins et al. [21] was used to determine the efflux capacity of the *P. aeruginosa* isolates. Ethidium-bromide (EtBr) intercalates between DNA and produces fluorescence under ultraviolet radiation. It also acts as a substrate for most bacteria efflux pumps. It is thus rapidly pumped out by an overexpressed efflux pump resulting in lack of fluorescence of the bacteria mass [21]. Isolates of *P. aeruginosa* were cultured in 5 mL of nutrient broth (Oxoid, London, UK) at 37°C for 24 h. The optical density (OD) of the cultures was adjusted to 0.5 McFarland. Mueller-Hinton agar (20 mL) plates containing ethidium bromide at concentrations of 0 to 2.5 mg/L were divided into sectors to form a cartwheel pattern. The OD adjusted cultures were swabbed on EtBr agar plates from the center to the edge of the plate. Each plate was swabbed with *P. aeruginosa* ATCC 27853 which served as a comparative control. The agar plates were then incubated for 16 h at 37°C and examined under an UV-transilluminator. The minimum concentration of EtBr (MC_EtBr_) that produced fluorescence of the bacteria mass after incubation for 24 h was used in determining the efflux capacity of the various isolates (Figure 3(c)). The capacity of each bacteria strain to efflux EtBr was ranked relative to the reference strain (*P. aeruginosa* ATCC 27853) by calculating the efflux capacity index (*σ*):

(1) $$\begin{array}{c} \sigma \;=\;\frac{{\text{MC}}_{\text{EtBr}\left(\text{MDR}\right)}-{\text{MC}}_{\text{EtBr}\left(R\right)}}{{\text{MC}}_{\text{EtBr}\left(\text{Ref}\right)}}. \end{array}$$

MC_EtBr(MDR)_ is minimum concentration of EtBr which produced fluorescence in MDR *P. aeruginosa.*MC_EtBr(*R*)_ is minimum concentration of EtBr which produced fluorescence in *P. aeruginosa* ATCC 27853. Efflux activity was ranked as very high (*σ*^∗∗∗∗^ = 7 to 9), high (*σ*^∗∗∗∗^ = 4 to 6), moderate (*σ*^∗∗∗∗^ = 1 to 3), and low (*σ*^∗∗∗∗^ = 0).

### 2.10. Detection of *β*-Lactamases and Aminoglycoside Modifying Enzyme Encoding Gene

Antibiotic resistance genes encoding some genetic variants of ESBLS (*bla*_*SHV1*_, *bla*_*TEM1*_, and *bla*_*CTMX*_), MBLS (*bla*_*VIM*_ and *bla*_*IMP*_), and aminoglycoside modifying enzymes (*aac (3)-IV*) were sought in the MDR isolates. Using the forward and reverse primers in Table 1, polymerase chain reaction was carried out using a thermal cycler in a final volume of 25 *μ*L containing 2 *μ*L of DNA template, 12.5 *μ*L of GoTaq Master Mix, 0.75 *μ*L of a 0.5 mM magnesium chloride, and 8.55 *μ*L of nuclease-free water. The DNA template was initially denatured at 94°C for 5 min, followed by 35 cycles of denaturation of 94°C for 30 sec and extension of 72°C for 1 min. Annealing temperatures for *bla*_*SHV1*_, *bla*_*TEM1*_, *bla*_*CTMX*_, *bla*_*VIM*_, *bla*_*IMP*_, and *aac (3)-IV* were 56°C for 1 min, 58°C for 1 min, 60°C for 30 sec, 51°C for 1 min, 55°C for 1 min, and 50°C for 1 min, respectively. Finally, the products were extended at 72°C for 10 min. The PCR products were examined on a 2% w/v agarose gel at 60 V for 120 min and visualized using a transilluminator.

### 2.11. Detection of Integrons in *P. aeruginosa* Isolates

Bacteria genomic or plasmid DNA may contain antibiotic resistance markers and gene cassettes (integrons) that encode resistance to several antimicrobial agents. In order to determine the prevalence of integrons in the isolates, amplifications of the 5′ conserved (GGCATCCAAGCGCAAG) and 3′conserved (AAGCAGACTTGACCTGA) regions of class 1 integron and class 2 integrons (with forward and reverse primers int2-F and reverse int2-R) were performed. The reaction conditions were an initial denaturation at 94°C for 5 min, further denaturation at 94°C for 1 min, annealing at 59.5°C for 1 min (class I integron), and 60°C for 1 min extension of 72°C (class 2 integron) for 4 min. The products were finally extended at 72°C for 10 min. 5 *μ*L of the amplicon was loaded into a 20-well 1.5% w/v agarose gel in 1X TAE (1 mM EDTA, 40 mM Tris-acetate) and run for 340 min at 65 V.

## 3. Results and Discussion

**Figure 4:**
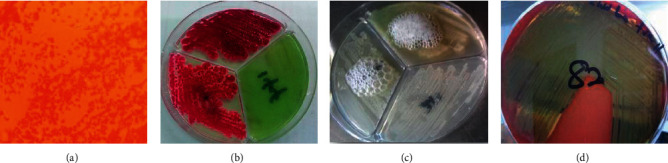
Biochemical characteristics of clinical, environmental, and poultry litter *P. aeruginosa* isolates. (a) Gram-negative unicellular rods of *Pseudomonas aeruginosa.* (b) Pigmentation of *P. aeruginosa* on *Pseudomonas* isolation agar (Alpha Biosciences, Maryland, USA). (c) Catalase production by *P. aeruginosa*. (d) *β*-Haemolysis of *P. aeruginosa* on blood agar.

A total of 87 *P. aeruginosa* isolates were confirmed from the clinical, environmental, and poultry litter samples. Morphologically, all the *P. aeruginosa* isolates were Gram-negative (Figure 4(a)) unicellular rods appearing as mucoid or nonmucoid colonies. The mucoid form is mainly due to alginate slime formation, which is presumed to play a role in colonization and virulence [27]. Identification of *P. aeruginosa* isolates included identifying the production of the soluble pigments (Figure 4(b)), pyocyanin (blue-green) [28, 29], fluorescein (greenish-yellow), pyorubin (red) [30], or pyomelanin (reddish-brown) [31, 32], growth at 42°C, and test for catalase production (Figure 4(c)) and *β*-haemolysis on blood agar (Figure 4(d)).

38 *P. aeruginosa* strains from clinical, environmental, and poultry litter sources which failed to produce characteristic pigments were identified through *opr*L gene amplification. This is consistent with reports by De Vos et al. [13] and Douraghi et al. [33] who illustrated the sensitivity of *opr*L outer membrane gene amplification in the identification of *P. aeruginosa* from both clinical and environmental sources.

There was low prevalence of *P. aeruginosa* (9.6%) in the samples collected from the various sources. Of the 900 samples, 162 isolates were identified through culture and biochemical characteristics (Figures 4(a)–4(d)). *P. aeruginosa* isolates from environmental (13.4%, *n* = 35/260) and clinical (12.9%, *n* = 47/364) samples were more prevalent compared to poultry litter (1.8%, *n* = 5/276) samples. Among the clinical samples, *P. aeruginosa* was highly prevalent in stool (39.7%, *n* = 31/78) than in urine samples (15.4%, *n* = 15/97) of patients (*p*=0.00077) (Table 2). This may be so because, aside from the resident *P. aeruginosa* colonization of the gastrointestinal tract, most ingested food, especially uncooked foods and slightly cooked foods, may be contaminated with *P. aeruginosa* and other pathogenic bacteria [34, 35].

This may increase its colonization of the gastrointestinal tract and hence its high prevalence in stool. All blood samples from the patients did not contain *P. aeruginosa.* The absence of *P. aeruginosa* in the blood samples, however, suggests that patients who took part in the study had no sepsis caused by *P. aeruginosa.* This finding is, however, contrary to a report by Opoku [36], from Komfo Anokye Teaching Hospital in Kumasi, Ghana, in which 11.83% of 187 samples obtained from blood of patients had *P. aeruginosa* colonization.

**Figure 5:**
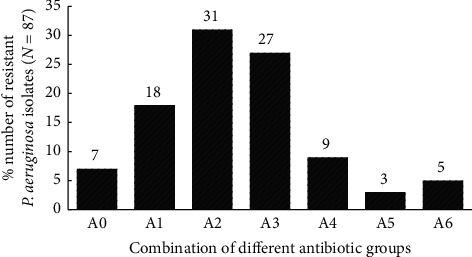
Number of resistant *P. aeruginosa* to the various antipseudomonal groups (quinolones, carbapenem, aminoglycosides, penicillin, cephalosporin, and monobactams). AO: no antibiotic group; A1: one antibiotic group; A2: two antibiotic groups; A3: three antibiotic groups; A4: four antibiotic groups; A5: five antibiotic groups; A6: six antibiotic groups.

7% of the confirmed *P. aeruginosa* strains (*n* = 6) of the isolates were sensitive to all antipseudomonal groups studied. 1, 16, and 21 poultry litter, clinical, and environmental isolates of *P. aeruginosa* were multidrug-resistant. From the study, nearly half (43.6%) of the isolates were resistant to at least three antipseudomonal groups (Figure 5). Comparing these findings to a study by Addo [37] who reported 13.04% MDR *in P. aeruginosa* isolates from wounds of patients comparably shows a surge in the number of MDR *P. aeruginosa* strains in the study isolates.

Enzymes that hydrolyze the *β*-lactam ring of antibiotics may be extended spectrum *β*-lactamases (ESBLS), metallo-*β*-lactamase (MBLS), inducible cephalosporinases (AmpC), or carbapenemases (KPC) [38]; *β*-lactamase inhibitors such as clavulanic acid inhibit *β*-lactamases produced in bacteria and thus may augment the activity of *β*-lactamase substrate antibiotics [39]. They can therefore be used for the detection of enzyme induction in a bacteria species using the double-disc synergy test (DDST). Addition of cloxacillin inhibits the activity of AmpC enzyme [16]. In the study, ESBLs were detected in 84.2% (*n* = 32) (Table 3) of the MDR *P. aeruginosa* isolates. Almost 88% (14/16) of the clinical and 80.9% (17/21) of the environmental MDR *P. aeruginosa* isolates produced ESBLs, indicating high prevalence of these enzymes in both clinical and environmental isolates. The MDR *P. aeruginosa* isolate from poultry litter was also found to produce ESBLs. About 5% of the study isolates produced no *β*-lactamase enzymes. Even though extended spectrum *β*-lactamase (ESBL) enzymes were prevalent in 84.2% of the MDR isolates, only 34.2% produced only ESBLs. This is similar to the findings of Newman et al. [8] who detected high ESBL production (90 to 98%) in Gram-negative isolates from the southern, middle, and northern sectors of Ghana. Metallo-*β*-lactamase enzymes (MBLs) and inducible AmpC *β*-lactamases were detected in 34.2% and 50% of the MDR isolates, respectively.

None of the *P. aeruginosa* isolates produced KPC type carbapenemase enzymes. Coproduction of ESBL, MBL and AmpC was predominant in the isolates with occurrence rates ranging from 21.1 to 44.7%. ESBL and AmpC coproduction was the most prevalent (44.7%) in the MDR isolate but the carbapenems (meropenem and imipenem) remained effective against these enzyme producers. ESBL producers were also susceptible to carbapenems as reported by Feglo and Opoku [40], and this confirms their role in the definitive treatment of ESBL producing bacteria strain infections. In a related report by Feglo and Opoku [40] on 187 *P. aeruginosa* isolates from Kumasi, 44.9% were AmpC producers and 21.9% were ESBL producers. These findings indicate an increase in both ESBL prevalence and MBL prevalence.

The prevalence of AmpC was relatively high compared to other study reports in Delhi, India (20.7%) [41], and MBL prevalence was similar to findings from Brazil which reported 36.4% ± 14.1 MBL occurrence in *P. aeruginosa* [42]. These findings could indicate geographical variations in the prevalence of AmpC producing *P. aeruginosa* and may be due to low usage of cephalosporins that induce production of this enzyme.

Genetic variants of common ESBL encoding antibiotic resistance genes (*bla*_*SHV*_, *bla*_*TEM*_, and *bla*_*CTMX*_) were not detected in any of the MDR isolates. The most common MBL encoding genes (*bla*_*IMP*_ and *bla*_*VIM*_) were also not detected in any of the isolates. Even though some common *β*-lactamase antibiotic-resistant genes were not detected, enzymes that are products of these genes were phenotypically detected in the isolates. This indicates that other *β*-lactamase encoding genes such as *bla*_PER_, *bla*_VEB_, *bla*_GES_, *bla*_PSE_, *bla*_SPM_, *bla*_GIM_, *bla*_AIM_, *bla*_NDM_, AmpC, and *bla*_OXA_, which have been found in *P. aeruginosa* from other geographical regions [38], may be responsible for the regulation of *β*-lactamase enzyme production. A similar study by Addo [37], in Korle Bu Teaching Hospital, Accra, and Regional Hospital, Koforidua, Ghana, also found no *bla*_VIM_ and *bla*_IMP_ carbapenemase encoding genes in *P. aeruginosa* strains from diabetic, burn, and cellulitic wounds of patients, suggesting their low prevalence in *P. aeruginosa* in Ghana.

Absence of these ESBL enzyme types could greatly enhance susceptibility of the *P. aeruginosa* strains to *β*-lactam substrates such as cephalosporins (ceftazidime, ceftriaxone, and cefepime), monobactams (aztreonam), carboxypenicillins, and ureidopenicillins. The absence of metallo-*β*-lactamase (carbapenemase) encoding genes, however, suggests that there was no impact of their enzyme variants to carbapenem (meropenem and imipenem) resistance in the *P. aeruginosa* strains isolated.

Circulation of enzyme groups of TEM, SHV, and CTX-M has, however, been detected in many *Escherichia coli* strains from patients in Komfo Anokye Teaching Hospital, Kumasi, Ghana [40]. IMP-1, IMP-6, IMP-9, VIM-1, VIM-3, and other metalloenzyme variants have also been identified in *P. aeruginosa* isolates from other countries like Japan, China, Singapore, Brazil, Italy, Greece, Taiwan, and Iran [43]*. P. aeruginosa* may gain resistance to aminoglycosides through a series of resistance mechanisms including enzymatic modification [44]. The presence of aminoglycoside resistance gene *AAC (3)-IV* (resistance to gentamycin, tobramycin, and netilmicin) was determined in both gentamycin-resistant and sensitive MDR *P*. aeruginosa. No *AAC (3)-IV* gene was detected in any of the MDR *P. aeruginosa* isolates. This rules out the impact of their enzyme variants in strains that were resistant to the studied aminoglycosides.

A total of 57.8% of the MDR isolates demonstrated moderate to very high efflux pump activity (Table 4). This may affirm an overactive efflux of antibiotics from the bacterial cell reducing the activity of the antipseudomonal antibiotics.

## 4. Conclusion

There was low prevalence (9.6%) of *P. aeruginosa* in the clinical, environmental, and poultry litter samples from the Ashanti Region of Ghana. There is, however, an appreciable surge in the number of MDR *P. aeruginosa* strains in the clinical and environmental samples. There was high prevalence of ESBLs (84.2%), MBLs (34.2%), and inducible cephalosporinase (AmpC) enzymes (50%) in the *P. aeruginosa* isolates from Ashanti Region of Ghana. Mobile genetic elements (class I integrons) were also highly prevalent (89.4%) in the *P. aeruginosa* strains. Antibiotics with activity against *P. aeruginosa* harboring these antibiotic degrading enzymes and resistance integrons should therefore be recommended for clinical treatment of related infections. Routine surveillance of new emerging MDR pathogenic bacteria strains should be undertaken in potential areas of high AMR selection. Also, some research focus should be directed towards the search for anti-infectives with marked activity against multidrug-resistant pathogenic bacteria.

## Figures and Tables

**Table 1:** Primer nucleotide sequences for detection of antibiotic-resistant genes.

Primer	Primer sequence (5′-3′)	Amplicon size (bp)	Annealing temperature (°C)	Homology	Reference/accession number
oprL-F	ATG GAA ATG CTG AAA TTC GGC	504	64	*P. aeruginosa* identification	[13]
oprL-R	CTT CTT CAG CTC GAC GCG ACG				
5′CS	GGCATCCAAGC GCAAG	Variable	59.5	Conserved region of class 1 integron	[22]
3′CS	AAG CAG ACT TGA CCT GA				
Int2-F	CACGGATATGCGACAAAAAGGT	788	60	Class 2 integron	[23]
Int2-R	GTAGCAAACGAGTGACGAAATG				
aac(3)-IV -F	GTGTGCTGCTGGTCCACAGC	627	50	Aminoglycoside acetyltransferase	[24]
aac(3)-IV-R	AGTTGACCCAGGGCTGTCGC				
SHV 1	GGG TTA TTC TTA TTT GTC GC	900	56	SHV ESBL	[25]
SHV 2	TTA GCG TTG CCA GTG CTC				
TEM1-F	ATG AGT ATT CAA CAT TTC CG	867	58	TEM ESBL	[25]
TEM1-R	CTG ACA GTT ACC AAT GCT TA				
CTMX-F	ATG TGC AGY ACC AGT AAR GTK ATG GC	593	60	CTMX ESBL	[26]
CTMX -R	TGG GTR AAR TAR GTS ACC AGA AYC AGC G				
VIM-F	ATG GTG TTT GGT CGC ATA TC	261	51	VIM MBL	[25]
VIM -R	TGG GCC ATT CAG CCA GAT C				
IMP -F	CTA CCG CAG AGT CTT TG	600	55	IMP MBL	[25]
IMP- R	AAC CAG TTT TGC CTT ACC AT				

**Table 2:** Sample screened and the number of *P. aeruginosa* isolates obtained.

Source of Sample	Total sample (*n* = 900)	Number of isolates (*n* = 87)	Percentage (%)
Clinical	364	47	12.9
Stool	78	31	39.7
Urine	97	15	15.4
Blood	100	0	0
Farm hands	89	1	1.1
Environmental	**260**	**35**	**13.4**

Sewage	96	12	12.5
Market floors	104	15	14.4
Others^a^	60	8	13.3
Poultry litter	**276**	**5**	**1.8**

Others^a^: environmental samples collected from market tables, community-based latrines, and water source.

**Table 3:** Prevalence of *β*-lactamase enzymes in MDR *P. aeruginosa* (*n* = 38).

Type of *β*-lactamase	Number of multidrug-resistant *P. aeruginosa* isolates										
	*n* (%)	CIP	LEV	MEM	IPM	CN	TIC	PIP	TIM	FEP	CAZ	ATM
No *β*-lactamase	2 (5.3%)	2		1	1	1	2	1	1	1	2	O
Only ESBL	13 (34.2%)	13	7	1	1	13	10	3	5	5	3	4
Only MBL	2 (5.3%)	1				2	1					2
Only AmpC	2 (5.3%)	2		1		1	1	1		1		2
ESBL + MBL	11 (28.9%)	8	5	1	2	8	10	2	3		1	2
ESBL + AmpC	17 (44.7%)	15	6	2	4	16	14	3	4	3	3	4
MBL + AmpC	8 (21.1%)	8	4	1	1	8	9	2	2		1	1
ESBL + MBL + AmpC	9 (23.7%)	8	5	1	2	8	9	2	2		1	1

CIP: ciprofloxacin; LEV: levofloxacin; MEM: meropenem; IPM: imipenem; CN: gentamycin; TIC: ticarcillin; TIM: ticarcillin/clavulanic acid; FEP: cefepime; ATM: aztreonam; CAZ: ceftazidime; PIP: piperacillin; N: number of multidrug-resistant isolates; ESBL: extended spectrum *β*-lactamase; MBL: metallo-*β*-lactamase; AmpC: cephalosporinase.

**Table 4:** Phenotypic and genotypic resistant determinants in *P. aeruginosa* isolates.

MDR strain	Source	ESBL	KPC	MBLS	Inducible AmpC	Efflux capacity index	Class 1 integron
PS_196_	E	+	−	−	+	0	+
PS_204_	E	−	−	+	−	0	+
PS_170_	E	+	−	−	−	0	+
PS_205_	E	+	−−	+	+	0	+
PS_195_	E	+	−	−	−	1	+
PS_197_	E	+	−	−	−	7	+
PS_168_	E	+	−	−	+	1	+
PS_185_	E	+	−	+	−	0	+
PS_155_	E	+	−	−	+	9	+
PS_137_	E	−	−	−	+	0	+
PS_139_	E	−	−	−	−	0	−
PS_133_	E	+	−	−	−	1	+
PS_123_	E	+	−	+	+	0	+
PS_113_	E	+	−	+	+	1	+
PS_112_	E	+	−	−	−	5	+
PS_108_	E	−	−	−	+	0	−
PS_102_	E	+	−	+	+	1	+
PS_167_	E	+	−	+	+	0	−
PS_105_	E	+	−	+	+	5	+
PS_109_	E	+	−	−	+	1	+
PS_111_	E	+	−	−	+	9	+
PS_231_	PL	+	−	−	−	1	+
PS_14_	S	+	−	−	+	0	−
PS_17_	S	+	−	+	+	4	+
PS_31_	S	+	−	−	+	1	+
PS_37_	S	−	−	+	−	0	+
PS_5_	S	+	−	−	−	0	+
PS_25_	S	+	−	+	+	5	+
PS_29_	S	+	−	−	−	0	+
PS_4_	S	+	−	−	−	0	+
PS_1_	S	+	−	+	+	7	+
PS_33_	S	+	−	−	−	1	+
PS_41_	S	+	−	+	−	0	+
PS_82_	U	+	−	−	−	1	+
PS_84_	U	+	−	−	−	0	+
PS_85_	U	−	−	−	−	1	+
PS_98_	U	+	−	−	+	3	+

+: present; ESBL: extended spectrum beta-lactamase; MBL: metallo-beta-lactamase; S: stool; U: urine; E: environment; PL: poultry litter.

## Data Availability

The data from the research are available in the University Institutional Repository KNUST Space through the following link: http://dspace.knust.edu.gh/handle/123456789/10233.

## Abbreviations

PCR: Polymerase chain reaction
ESBL: Extended spectrum *β*-lactamase
MBL: Metallo-*β*-lactamase
MDR: Multidrug-resistant.
